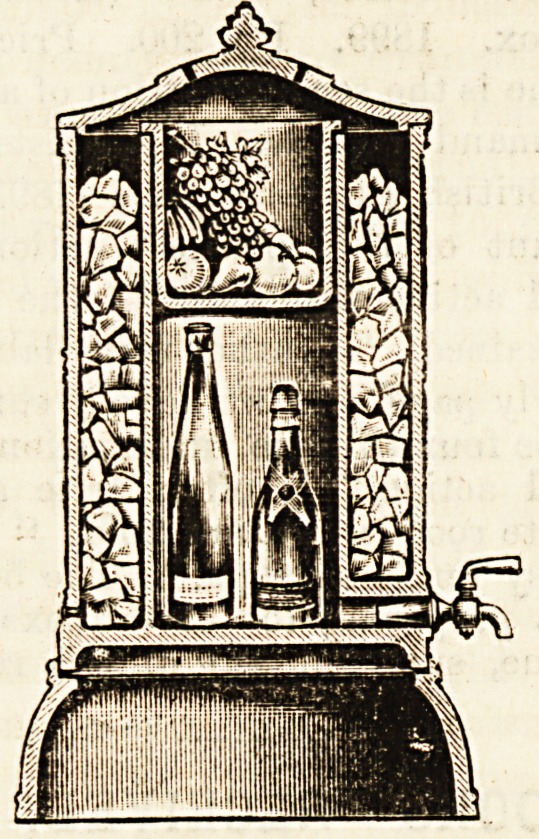# New Appliances and Things Medical

**Published:** 1899-07-15

**Authors:** 


### NEW APPLIANCES AND THINGS MEDICAL.

[We shall be glad to receive, at our Office, 28 & 29, Southampton Street, Strand, London, W.C., from the manufacturers, specimens of all new preparations and appliances which may be brought out from time to time.]

### THE "ICELAND" PATENT COMBINATION COOLER AND FILTER.

(Morrison, Ingram, and Co., Limited, Hygeia Works, Hadfield Street, Cornbrook, Manchester.)

This novel invention, which may be used either as a convenient refrigerator or combined filter and cooler, is made of porcelain or fine caneware. It consists of a double chamber, with a contained space for ice or other freezing mixture. When used as a filter the filtering medium consists of special carbon, which can be withdrawn, cleaned, and replaced with the least possible trouble. Every part of the filter is easily accessible, and there is no complicated mechanism to get out of order. The tap for drawing off the water is of the Hygeia white metal variety. When in use as a cooler the filtering tray can be withdrawn and the central chamber utilised for cooling wine or other fluids and comestibles. The "Iceland" can be procured in various sizes, and for sideboard use in elegant form. The simplicity, cleanliness, and practical nature of the invention will be appreciated by all those who give it a trial.

### NON-FARINACEOUS INFANTS' FOOD.

(Callard and Co., 65, Regent Street, W.)

Unfortunately, at the present day nurses and doctors often find it impossible to bring up an infant satisfactorily on diluted or modified cow's milk. For this reason various prepared foods have won considerable popularity, since when they are mixed with diluted milk they not only render the clotting in the stomach lighter, more flocculent and easily digested, but when correctly prepared they brink the diluted milk up to the proper standard of human milk. Callard's Non-Farinaceous Food in these respects appears to fulfil all the conditions of an ideal food. As its name implies, it contains no unconverted starch, but a proper proportion of proteids, dextrin, sugar, fat, and phosphates, all in a readily-absorbable form. By following out the directions provided with each tin of the food, an absolutely correct and palatable infant's food can be made by the most ignorant with the minimum of trouble and a maximum of certainty.

### PATENT REFINED SPARKLING GELATINE.

(J. and G. Cox, Edinburgh. London Office: Eastcheap Buildings, E.C.)

The value of an absolutely pure jelly for sick-room use cannot be over-estimated. Owing to the time and trouble necessary to prepare a home-made calf-foot jelly, doubtful or worthless manufactured substitutes are often resorted to by cook or nurse. The old superstition that glorified the home-made calf-foot jelly, and imprinted on it a nutritive value far beyond that of other forms of pure gelatine, is, in fact, becoming a discredited old wife's tale; and we now know that, provided gelatine is pure, one form has no more nutritive value than another. The manufacture of pure gelatine is not, however, one of the easiest processes in the world, but we believe that in Cox's gelatine the public and nursing world have an article of very high value, and one on which they can absolutely depend. As a basis for all sorts of jellies, whether sweet or otherwise, it will be found not only economical, but when of the requisite degree of dilution beautifully sparkling, firm, and soluble in the mouth.

### SAFETY BRONCHITIS KETTLE.

(Maw, Son, and Thompson, 7 to 12, Aldersgate Street, E.C.)

That certain dangers attach to the use of the ordinary bronchitis kettle has more than once been unhappily demonstrated. For instance, it is easy to overfill the kettle, and when the pressure of steam within reaches a certain degree, water instead of steam is forced out of the egress spout—to the imminent danger of the patient. In the new safety kettle the level to which it can be filled through the hole of ingress is below the level of the egress hole to which the spout is soldered. Considering that this kettle is being placed on the market at a price little or no higher than the ordinary variety, it is the duty of persons purchasing a bronchitis kettle to see that this kind is supplied.

## Figures and Tables

**Figure f1:**